# EGFR Inhibition Abrogates Leiomyosarcoma Cell Chemoresistance through Inactivation of Survival Pathways and Impairment of CSC Potential

**DOI:** 10.1371/journal.pone.0046891

**Published:** 2012-10-08

**Authors:** Giovanni Sette, Valentina Salvati, Lorenzo Memeo, Katia Fecchi, Cristina Colarossi, Paola Di Matteo, Michele Signore, Mauro Biffoni, Vito D’Andrea, Enrico De Antoni, Vincenzo Canzonieri, Ruggero De Maria, Adriana Eramo

**Affiliations:** 1 Department of Hematology, Oncology and Molecular Medicine, Istituto Superiore di Sanità, Rome, Italy; 2 Mediterranean Institute of Oncology, Catania, Italy; 3 Department of Surgical Sciences, La Sapienza University of Rome, Italy; 4 Division of Pathology, Centro di Riferimento Oncologico, Istituto Nazionale Tumori, Aviano, Italy; 5 Regina Elena National Cancer Institute, Rome, Italy; University of Pittsburgh Cancer Institute, United States of America

## Abstract

**Background:**

Tumor cells with stem-like phenotype and properties, known as cancer stem cells (CSC), have been identified in most solid tumors and are presumed to be responsible for driving tumor initiation, chemoresistance, relapse, or metastasis. A subpopulation of cells with increased stem-like potential has also been identified within sarcomas. These cells are endowed with increased tumorigenic potential, chemoresistance, expression of embryonic markers, and side population(SP) phenotype. Leiomyosarcomas (LMS) are soft tissue sarcomas presumably arising from undifferentiated cells of mesenchymal origin, the Mesenchymal Stem Cells (MSC). Frequent recurrence of LMS and chemoresistance of relapsed patients may likely result from the failure to target CSC. Therefore, therapeutic cues coming from the cancer stem cell (CSC) field may drastically improve patient outcome.

**Methodology/Principal Findings:**

We expanded LMS stem-like cells from patient samples *in vitro* and examined the possibility to counteract LMS malignancy through a stem-like cell effective approach. LMS stem-like cells were *in vitro* expanded both as “tumor spheres” and as “monolayers” in Mesenchymal Stem Cell (MSC) conditions. LMS stem-like cells displayed MSC phenotype, higher SP fraction, and increased drug-extrusion, extended proliferation potential, self-renewal, and multiple differentiation ability. They were chemoresistant, highly tumorigenic, and faithfully reproduced the patient tumor in mice. Such cells displayed activation of EGFR/AKT/MAPK pathways, suggesting a possibility in overcoming their chemoresistance through EGFR blockade. IRESSA plus Vincristine treatment determined pathway inactivation, impairment of SP phenotype, high cytotoxicity *in vitro* and strong antitumor activity in stem-like cell-generated patient-like xenografts, targeting both stem-like and differentiated cells.

**Conclusions/Significance:**

EGFR blockade combined with vincristine determines stem-like cell effective antitumor activity *in vitro* and *in vivo* against LMS, thus providing a potential therapy for LMS patients.

## Introduction

Soft tissue sarcomas constitute a heterogeneous group of rare tumors, accounting for 1% of adult neoplasias and 10% of pediatric malignancies [1]. Leiomyosarcomas (LMS), representing 5 to 10% of all soft tissue sarcomas, are malignant soft tissue tumors with smooth muscle differentiation. Similarly to other types of sarcomas, they most probably arise from the undifferentiated cells of mesenchymal origin, the Mesenchymal stem cells (MSC) [1], [2], [3], [4], [5]. Patients are treated with wide surgical excision followed by radiotherapy in most cases [2], [3]. Despite this local treatment, the rate of metastatic relapse is about 40% at the 5 year follow up [6]. Over the last few years, adjuvant chemotherapy has demonstrated increased survival benefit for treated patients. However, the outcome remains poor, and patients with relapsed disease remain largely incurable. In the past, all subtypes of soft tissue sarcomas were merged into the same retrospective analyses, thus reporting a global weak response to chemotherapy in clinical trials and a median survival generally lower than 1 year. More recently, the analysis of selected histological variants exposed to specific histology-tailored treatments, have demonstrated a better response rate [7], [8], [9], [10]. These retrospective analyses and subsequent prospective studies documented clinical benefit for LMS patients treated with doxorubicin, gemcitabine/docetaxel combination regimens, temozolomide and the recently introduced biological agent trabectedin [7], [11], [12], [13], [14]. However, the clinical outcome in relapsed patients remains poor, calling for innovative drugs directed against key molecular targets involved in tumor development and progression. The AKT-mTOR pathway activation has been identified as a key event for the development of LMS [15]. Therefore, targeting key elements of these survival pathways may lead to more effective antitumor strategies against LMS. In addition, even targeting deregulated oncogenic and survival pathways might not be sufficient to achieve tumor cell death, since other mechanisms may contribute to chemoresistance of soft tissue sarcomas, including their marked ability to limit intracellular accumulation of anti-neoplastic agents by active drug extrusion [16]. Increased chemoresistance and survival, as well as elevated membrane transporter activity, has been associated to stem-like cells. Therefore, innovative hints for the battle against solid tumors may emerge from cancer stem cells (CSC) research [17]. Important studies have highlighted a key role of CSC in development, maintenance, metastasis, chemoresistance and relapse of solid tumors, indicating these undifferentiated transformed stem cells as primary targets for more effective anti-cancer therapies [18], [19], [20], [21], [22], [23], [24]. CSC have been recently identified in bone sarcoma cell lines as a small subpopulation of cells capable of forming suspended spherical, clonal colonies in anchorage independent, serum-starved conditions, and expressing embryonic and MSC antigens [25], [26], [27]. Another study identified sarcoma initiating-cells in different types of sarcomas based on their ability to extrude Hoechst 33342 dye, determining their Side Population (SP) phenotype [28]. SP cells displayed high tumorigenic potential, while the proportion of SP cells correlated with tumor aggressiveness, suggesting that interfering with the SP phenotype or with other CSC properties could constitute a strategy to counteract soft tissue sarcoma aggressiveness [28], [29]. These reports highlighted the possibility to investigate the existence and nature of CSCs in different types of soft tissue sarcomas, paving the way for potential identifycation of innovative therapeutic targets for these deadly cancers [29]. Here, we exploit two different technologies to isolate and expand LMS-CSCs. The availability of exponentially growing CSCs, allowed us to obtain *in vitro* characterization of the tumorigenic population and develop preclinical therapeutic models to investigate more effective treatments for LMS patients.

## Materials and Methods

### Ethics Statement

Tumor samples were obtained in accordance with consent procedures approved by the Internal Review Board of Department of Surgical Sciences, Division of General Surgery, La Sapienza University, Rome, Italy and by the International Review Board for Research on solid tumors (Research Line 4) of Division of Pathology, Centro di Riferimento Oncologico, Istituto Nazionale Tumori, Aviano, Italy. All patients agreed to participate in the study and signed an informed consent form.

According to the Legislative Decree 116/92 which has implemented in Italy the European Directive 86/609/EEC on laboratory animal protection, the research protocol “Analysis of effectiveness and tolerability of anti-tumor therapeutic agents in mice carrying cancer stem cell-derived tumors” (Principal Investigator Dr. Adriana Eramo) has been approved by the Service for Biotechnology and Animal Welfare of the Istituto Superiore di Sanità and authorized by the Italian Ministry of Health (Decree n° 217/2010-B). The animals used in the above mentioned research protocol have been housed and treated according to Legislative Decree 116/92 guidelines, and animal welfare was routinely checked by veterinarians from the Service for Biotechnology and Animal Welfare.

### Isolation and Culture of Leiomyosarcoma Stem and Differentiated Cells

Tissue dissociation and culture of cell suspension were obtained as previously described for tumor sphere obtainment [18], [19]. Alternatively, cell growth of undifferentiated LMS cells as adherent cultures was obtained in culture conditions used for MSC (α-MEM medium supplemented with 20% MSC-suitable FBS, Stem Cell Technologies). Differentiated primary cultures were obtained as adherent monolayers following growth conditions suitable for sarcoma cell lines. Cells obtained from freshly dissociated tumors were cultivated in DMEM medium supplemented with 10% FBS, or alternatively tumor spheres were dissociated and cultured under the same conditions.

### Self-renewal Assay

To evaluate the fraction of self renewing cells, undifferentiated or differentiated LMS cells or MSC were plated on 96-well plates at a concentration of a single cell per well. Wells containing either none or more than one cell were excluded from the analysis. Colonies were counted after 4 weeks. For secondary sphere formation assay, single spheres obtained from primary cloning were dissociated in single cells, re-plated and treated as for primary spheres.

### Mesenchymal Differentiation of LMS Stem like-cells

LMS stem-like cell differentiation toward mesenchymal lineages was obtained using hMSC differentiation bullet Kit-Osteogenic, -Chondrogenic or –Adipogenic following the manufacturer’s instructions (Lonza, East Rutherford, NJ, USA). The acquisition of differentiation markers was evaluated by visible chemical/stain reaction with Oil red O (Sigma-Aldrich, St. Louis, Mo, USA) for adipogenic, with Alkaline Phosphatase substrate kit III (Vector Laboratories, Burlingame, CA, USA) for osteogenic or by alcian blue-PAS staining of differentiated cell pellet after cytoinclusion.

### Flow Cytometry, SP and Drug Efflux Ability Assay

Flow cytometry antibodies used were: PE-conjugated anti CD105 from R&D, PE-conjugated anti CD146, CD166 and CD73, FITC-conjugated anti CD44, PE-cy5 conjugated anti c-Kit (all from BD) and Smooth Muscle Actin (Dako). Stained cells were analyzed with FACSCanto (BD). For SP, LMS stem-like cells and LMS differentiated cells (1×10^5^) were incubated for 90 min with 10 µg/ml Hoechst 33342 (Molecular Probes) dye alone or with 50 µM Verapamil (Sigma), counterstained with 100 µg/ml 7AAD to exclude non-viable cells and analyzed with a dual wavelength analysis (blue, 424–444 nm; red, 675 nm) with excitation with 350 nM UV light (FACS LSRII, BD). For drug efflux evaluation, intracellular doxorubicin retention assay was performed as previously described. Briefly, 2 hours of exposure to 5 µM doxorubicin (uptake) was followed by washing and overnight incubation in fresh culture medium (efflux). Intracellular doxorubicin-linked fluorescence was measured by flow cytometry.

### Cell Cycle Analysis

Cells (1×10^5^) were washed with PBS and re-suspended 0.1% sodium citrate, pH 7.4/0.1% Triton X100, containing 100 µg/ml propidium iodide and 200 µg/ml RnaseA. After 2 hrs of incubation at 4°C, samples were analyzed with FACSCanto (BD).

### Immunohistochemistry

Formalin-fixed paraffin-embedded 3 micron tissue sections were deparaffinized in xylene and rehydrated in a graded series of alcohol. The tissue sections were automatically stained for Smooth Muscle Actin (Roche Diagnostics, clone 1A4), Ki-67 (Roche Diagnostics, clone 30-9), on Benchmarker XT (Roche Diagnostics) following the manufacturer’s directions. Immunohistochemical stainings for pEGFR (clone 53A5, Cell signaling technology), EGF (polyclonal, Novus Biological), myogenin, myoglobin, vimentin, muscle specific actin HHF35 and caldesmon (all from Roche) were performed using avidin-biotin-peroxidase complex (UltraTek HRP, Scy Tek Laboratories). Slides were counterstained with hematoxylin and permanently mounted.

### Western Blot and Reverse Phase Phosphoproteomic Array (RPPA)

Proteins were resolved on 4–12% polyacrylamide gels (Invitrogen) and transferred to nitrocellulose membranes. Rabbit polyclonal anti-Phospho-Akt (Ser473), -Phospho-S6 (Ser240/244), -Phospho EGFR (Tyr 1068) were purchased from Cell Signaling. Mouse monoclonal anti-PTEN was purchased from BD, anti-Phospho-ERK (clone E-4), rabbit polyclonal anti-Bcl-2 and anti-BAX (N20) were from Santa Cruz. Mouse monoclonal β-actin clone (A5441) was by Sigma.

RPPA was performed as previously described [30]. Briefly, for RPPA analysis samples were lysed using T-PER (Tissue Protein Extraction Reagent, Thermo Scientific, Waltham, MA, USA) and diluted up to 0.5 mg/mL with Novex Tris-Glycine SDS Sample Buffer 2X (Invitrogen Corporation, Carlsbad, CA, USA). Subsequently, protein lysates were spotted in a two-fold 5 point dilution curve onto nitrocellulose-coated microscope slides via Aushon Arrayer 2470 (Billerica, MA, USA). Then each slide underwent incubation with a single validated primary antibody using DAKO Autostainer Plus (DAKO Corporation, Glostrup, Denmark). Signal amplification was performed by using DAKO CSA kit (Catalyzed Signal Amplification, DAKO) and diamminobenzidine was used for colorimetric detection of the primary antibody signal. Total protein quantification was performed using Sypro Ruby Protein Staining solution (Invitrogen) and total protein slides were scanned using a Vidar Revolution 4200 microarray scanner (Vidar Systems Corporation, Herndon, VA, USA). Antibody slides were scanned using a flatbed scanner and raw images were loaded into MicroVigene software (VigeneTech Inc., Boston, MA, USA) for secondary antibody subtraction and normalization to total protein.

### Drug Treatment and Proliferation Assay

Cells (2.5×10^3^) were seeded in 96-well plates and exposed for 72hours to: Vincristine 15 nM, Doxorubicin 100 nM, Temozolomide 250 µM, Dacarbazine 5 µg/ml, Etoposide 10 µg/ml, Gemcitabine 250 µM, Docetaxel 1 µg/ML, IRESSA 10 µM. Cell viability was detected with Cell Titer Glo (Promega). For cell proliferation assay cells were counted by Trypan Blue exclusion.

### *In vivo* Leiomyosarcoma Xenografts Generation and Mice Treatments

Cell suspensions were mixed 1∶1 with growth factor reduced Matrigel (BD) and injected subcutaneously in the flanks of four week-old female NOD-SCID mice (Charles River). For drug treatment, when tumors reached a mean of 0.5 cm diameter, mice were assigned into 4 treatment groups: a) control (vehicles only); b) IRESSA(100 mg/kg/5 days on and 2 days of/gavage); c) Vincristine (1 mg/kg/biweekly/I.P.); d) IRESSA+Vincristine (given concurrently with the same schedules and doses as the single drugs were given). At the end of treatments tumors were collected, fixed in formalin and embedded in paraffin for IHC and TUNEL assay. In each group two mice were left alive in order to check tumor growth at the end of treatment. IHC was performed as indicated above. Apoptotic cells were detected by TUNEL assay (Roche) following the manufacturer’s instructions.

## Results

### Isolation and Phenotypic Characterization of LMS Undifferentiated Cells

**Figure 1:**
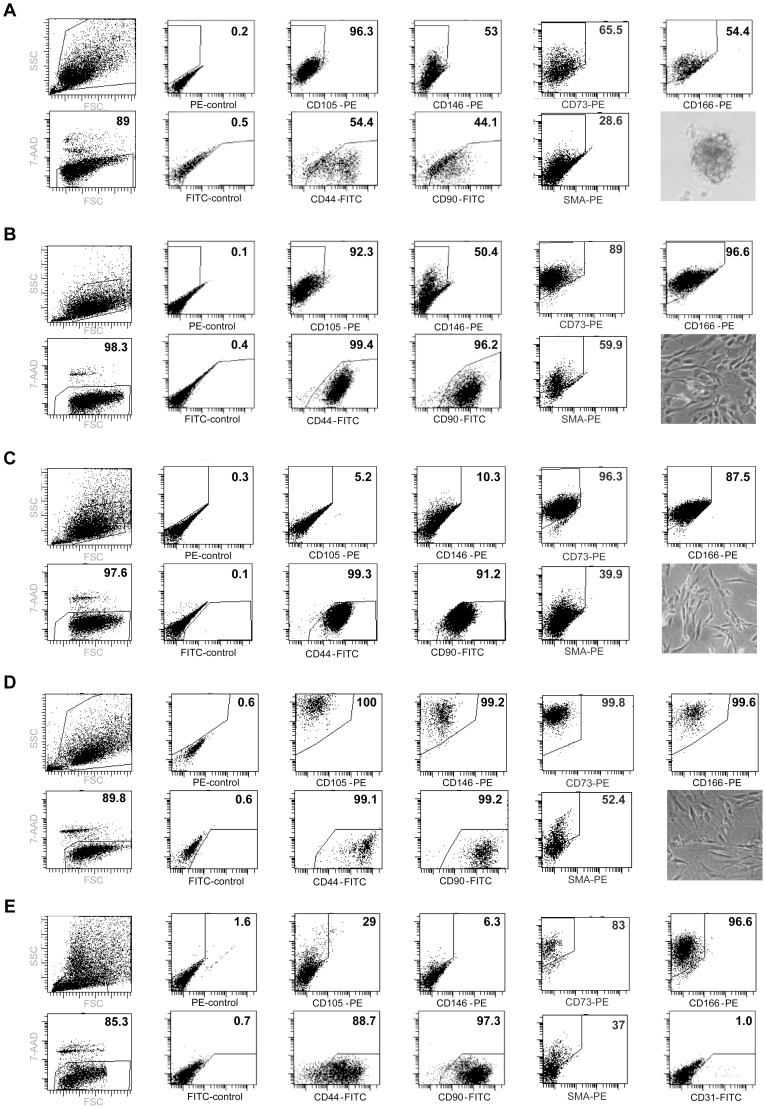
Undifferentiated LMS cells display a Mesenchymal Stem Cell (MSC)-phenotype. Flow cytometric detection of the indicated antigens in undifferentiated LMS cells isolated as tumors spheres (A), as adherent cells in MSC-culture conditions (B), as differentiated cells obtained in standard culture conditions (C), in non tumoral mesenchymal stem cells (D) or in fresh tumor cells (E). Representative FACS dot plots (SSC vs FSC), 7-AAD staining and images of the corresponding cells are reported for each condition.

*In vitro* culture and expansion of sarcoma CSCs have been obtained only from established cell lines. We investigated the possibility to isolate and expand the small fraction of undifferentiated tumor cells from a very rare form of LMS, generating long term cultures highly enriched with these cells. Tumor cells from enzymatically dissociated testicular LMS were cultured in conditions that we and others have previously demonstrated to enrich for CSC of various types as “tumor spheres”. After approximately 1 month of culture, surviving cells started to grow as floating cellular clusters called “sarcospheres” (Figure 1A, right bottom). In addition to the standard stem cell culture methodology, based on the assumption that the tumorigenic cells in sarcomas should consist in transformed undifferentiated cells of mesenchymal origin, i.e. transformed MSC, we evaluated the possibility to isolate these undifferentiated tumor cells in the same culture conditions widely used for non-transformed MSCs [31], [32]. Under these conditions, we obtained a monolayer of cells resembling MSCs for morphology, after few weeks of culture (Figure 1B, right bottom). At the same time, we cultured a fraction of the same cells in standard conditions for cell lines in order to obtain differentiated tumor cells. Under these conditions cells transiently proliferated as adherent monolayers for a few weeks. Subsequently they became quiescent, acquired the morphology of senescent cells and ultimately died, as expected for primary differentiated cells. In order to validate the nature of both sphere- and adherent-cultures, we first performed their immunophenotypical analysis, and compared it with differentiated LMS cells obtained under standard conditions (Figure 1 C) and with non tumoral MSC, used as control for undifferentiated mesenchymal cells (Figure 1D). We investigated the expression of markers associated with mesenchymal stem and/or differentiated cells. Both sphere-forming cells (Figure 1 A) and adherent cells (Figure 1 B) expressed high levels of CD105, CD146, CD166, CD44 and CD90 confirming a MSC phenotype [33], [34]. However, although CD166, CD44 and CD90 were abundantly present in all three cell culture types analyzed, as expected for these broadly expressed mesenchymal antigens, the MSC-restricted markers CD146 and CD105 were considerably up-regulated in both sarco-spheres (Figure 1A) and adherent MSC-like cultures (Figure 1B) in comparison with LMS cells obtained under standard conditions (Figure 1C). Freshly isolated tumor cells (Figure 1E) and cells grown in standard cultures (Figure 1C) displayed a similar antigenic pattern. In addition, both sarcospheres and adherent undifferentiated cells expressed high levels of the MSC marker CD73, similarly to non tumoral MSC. This marker was also expressed by the fresh tumor cells and cells cultured under standard conditions. As expected, the Smooth Muscle Actin (SMA) was expressed in all cell populations analyzed in agreement with the smooth muscle histology of LMS, and with previous reports showing SMA expression in non tumoral MSC (Figure 1) [35].

These results suggest that under standard conditions, we could obtain short term cultures of differentiated cells, while under appropriate stem cell conditions, both experimental strategies used (spheres and MSC-like monolayers) were suitable to select and expand the small fraction of CD105/CD146 positive undifferentiated LMS cells with stem-like phenotypes, determining their marked enrichment within the cell cultures [34].

### *In vitro* Cancer Stem Cell Potential of LMS Undifferentiated Cells

We next investigated whether *in vitro* expanded LMS cells were endowed with the stem cell-associated properties to extensively proliferate, self-renew, differentiate toward multiple lineages and extrude fluorescent dyes or drugs, as side population (SP) cells.

**Figure 2:**
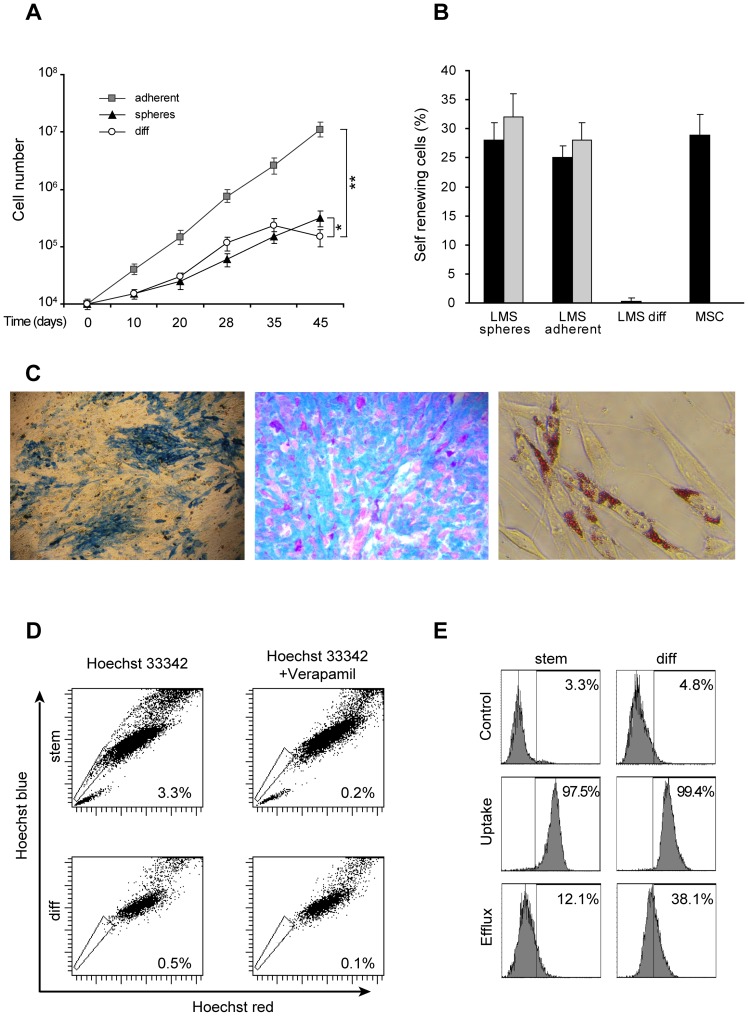
Undifferentiated LMS cells display stem cell potential in vitro. A) Growth curve of undifferentiated LMS cells isolated as spheres or adherent cultures as indicated, and of differentiated tumor cells (diff) obtained under standard conditions. The values represent mean +/− SD of three independent experiments. Student’ s T test was used to determine p-value. * p<0,05; **p<0,01. B) Self renewing ability (percentage of clonogenic cells) of undifferentiated (LMS spheres and LMS adherent) differentiated (LMS diff) LMS cells and MSC. Black bars represent primary assay, grey bars are secondary assays. Mean ± SD of 3 independent experiments is shown. C) Mesenchymal differentiation of LMS stem like-cells: (left) Osteogenic differentiation (Alcaline Phosphatase activity), (middle) Chondrogenic differentiation (Alcian-PAS), (right) Adipogenic differentiation (Oil-red-O). D) Flow cytometry analysis of Hoechst 33342-stained cells. The percentage of side population (SP) cells (gated) is indicated. E) Cytoﬂuorimetric profile of LMS undifferentiated (stem) and differentiated (diff) cells, untreated (Control), after O/N exposure to 5 µM doxorubicin (Uptake), or after 2 h of drug treatment followed by washing and overnight incubation in fresh medium (Efﬂux).

Both spheres and adherent cultures could be maintained *in vitro* for several months, by passaging them approximately once a week indicating that they were endowed with the CSC-associated property of extensive proliferative capacity (Figure 2A shows the growth rate of cells after being kept in culture for more than 20 passages). The growth rate was particularly high in the adherent cultures, suggesting that the medium developed for MSC is particularly suitable for their expansion (Figure 2A). In contrast, primary cultures obtained from freshly dissociated tumor cells, grown under standard conditions for cell lines, showed limited proliferation potential (Figure 2A). These results suggest that while the long term expansion of immature tumor cells is efficient under stem cell suitable conditions, obtaining a cell line from the same sample is far more challenging under standard conditions. Undifferentiated LMS were highly clonogenic in both primary and secondary limiting dilution assay, whereas differentiated LMS cells were unable to self renew, as indicated by the absence of colonies obtained in the same assay (Figure 2B). In addition, the percentage of clonogenic cells was similar in undifferentiated LMS and MSC cultures, indicating that these culture conditions resulted in a similar enrichment both for normal and transformed undifferentiated mesenchymal cells. Secondary sphere formation represents the best surrogate assay to assess self-renewal capacity *in vitro*. Thus, both types of undifferentiated LMS cultures were enriched with cells endowed with self renewal ability (Figure 2B).

We next investigated whether the LMS undifferentiated cells behaved as their putative non-tumoral counterpart, the MSC, in terms of multiple differentiation ability. We evaluated their ability to differentiate toward the 3 mesenchymal lineages adipogenic, chondrogenic and osteogenic, under appropriate culture conditions. Differentiation was obtained with high efficiency as demonstrated (Figure 2C) by the relatively high fraction of cells that stained positive for each specific differentiation marker used: the activity of alcaline phosphatase (osteo), staining with Blue Alcian Pas (chondro) or Oil-red-O positivity (adipo). Based on the reported high tumor-initiating potential of SP cells isolated from mesenchymal tumors, we next investigated whether the undifferentiated LMS cultures possessed a higher fraction of cells with SP phenotype, compared with their differentiated counterpart. In contrast to primary cultures, undifferentiated LMS cells displayed a higher number of SP cells (Figure 2D). The SP phenotype is a stem cell-feature that reflects an increased activity of ABC transporters, enhancing the ability of tumor cells to extrude drugs. As shown in Figure 2E, fluorescent doxorubicin was massively removed from undifferentiated LMS cells, whereas primary differentiated cells retained the drug-related fluorescence to a higher extent (Figure 2E).

These results suggest that undifferentiated LMS cells grown in stem cell media display some features shared by stem cells, such as surface markers, SP phenotype, unlimited proliferation, increased self renewal and multipotency. Thus, fulfiling all the *in vitro* requisites for potential LMS-CSC.

### Tumorigenic Potential of LMS Undifferentiated Cells and Recapitulation of Patient Tumor Histology and Differentiation Grade

**Figure 3:**
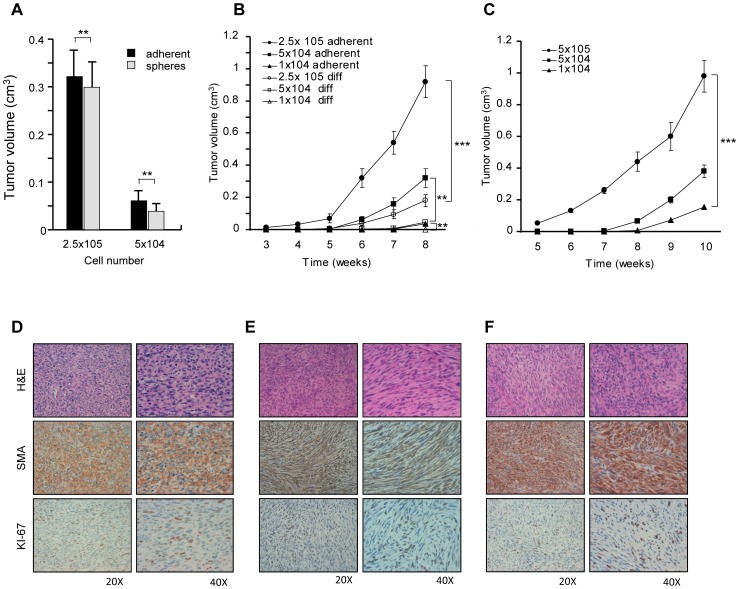
Undifferentiated LMS cells are highly tumorigenic and reproduce the human tumor in immunocompromised mice. A) Tumor volumes of xenografts generated by injection of spheres or adherent undifferentiated LMS cells (6 weeks post-injection). Mean ± SD of 3 independent experiments is shown. ** p<0,01. B) Tumor growth rate of undifferentiated (adherent) or differentiated (diff) LMS cells injected subcutaneously in NOD-SCID mice at the indicated cell doses. Mean ± SD of 3 independent experiments is shown. ** p<0,01, ***p<0,001. (C) Tumor growth rate of secondary tumors injected subcutaneously in NOD-SCID mice at the indicated cell doses. Mean ± SD of 3 independent experiments is shown. ***p<0,001. D) Hematoxylin and eosin (H&E) or immunohistochemistry for the indicated antigens performed on patient tumor (D), tumor generated by subcutaneous injection of LMS spheres (E) or adherent undifferentiated cells (F). The original magnification for each image is indicated.

In order to investigate whether spheres and adherent undifferentiated LMS cells could be considered LMS stem-like cells, we evaluated whether they possessed stem cell properties *in vivo*. Therefore, we investigated their tumorigenic potential and their ability to recapitulate the original patient tumor in animal models. Subcutaneous injection of undifferentiated LMS cells in NOD-SCID mice resulted in efficient and cell number-dependent tumor growth (Figure 3A and 3B). Both types of undifferentiated cultures were highly tumorigenic, with tumor growth being slightly more rapid for adherent LMS cells (Figure 3A). In contrast, the level of tumorigenic activity of primary differentiated cells was considerably lower as tumors generated from the injection of differentiated cells grew very slowly and with low efficiency when low numbers of cells were injected (Figure 3B and Figure S1A).

We compared the expression of the MSC markers CD146 and CD105 in the parental and xenograft tumors. Even though these markers were considerably upregulated in the undifferentiated LMS cells, the resulting tumor xenografts showed a similar frequency like the parental tumor (Figure S1B). These results suggest that such LMS stem-like cells were able to generate a patient tumor-like differentiated progeny *in vivo*. LMS cells obtained from these primary tumors were able to rapidly and efficiently form secondary tumors in a dose dependent manner (Figure 3C). This demonstrates that CSCs self-renewed *in vivo* maintaining their ability to generate new tumors. Secondary tumor growth rate was lower than that of CSC-generated primary tumors, as expected for the injection of a cell population composed mostly by differentiated cells (those obtained from dissociation of primary tumor).

Mice xenografts were collected and analyzed for similarity with the original patient tumor in terms of morphology and immunohistochemistry. Both sphere- (Figure 3E) and adherent culture- (Figure 3F) generated xenografts highly resembled the patient tumor (Figure 3D), as indicated by hematoxylin and eosin staining, expression of the LMS diagnostic marker Smooth Muscle Actin (SMA), caldesmon, HHF35 and vimentin (Figure 3D–F and Figure S2A). As expected, the Rabdhomyosarcoma markers Myogenin and Myoglobin were not expressed, further demonstrating the ability of the LMS undifferenitated cells to differentiate *in vivo* into a sarcoma of the same subtype as the patient tumor (Figure S2A). Additionally, the equal frequency of Ki67^+^ cells (between 18 and 20% in all samples as evaluated by the observation of 10 high power fields), suggests an equal growth rate of the different tumors (Figure3D–F). Importantly, the histology of secondary tumors and tumors generated by differentiated cells shared the same features as the primary xenograft and the patient tumor (Figure S2C). Based on these results, both sphere and adherent cultures of LMS stem-like cells appear highly tumorigenic and suitable for preclinical experiments. Given that the MSC growth conditions provided a system of more rapidly growing cells, adherent LMS stem-like were employed for most of the subsequent studies. Although all the *in vivo* experiments were performed in the presence of matrigel, cells injected in the absence of matrigel retained their tumorigenic ability, which was only delayed by the absence of a supportive microenvironment for initial growth (Figure S2B).

### Chemoresistance of LMS Stem Cells

**Figure 4:**
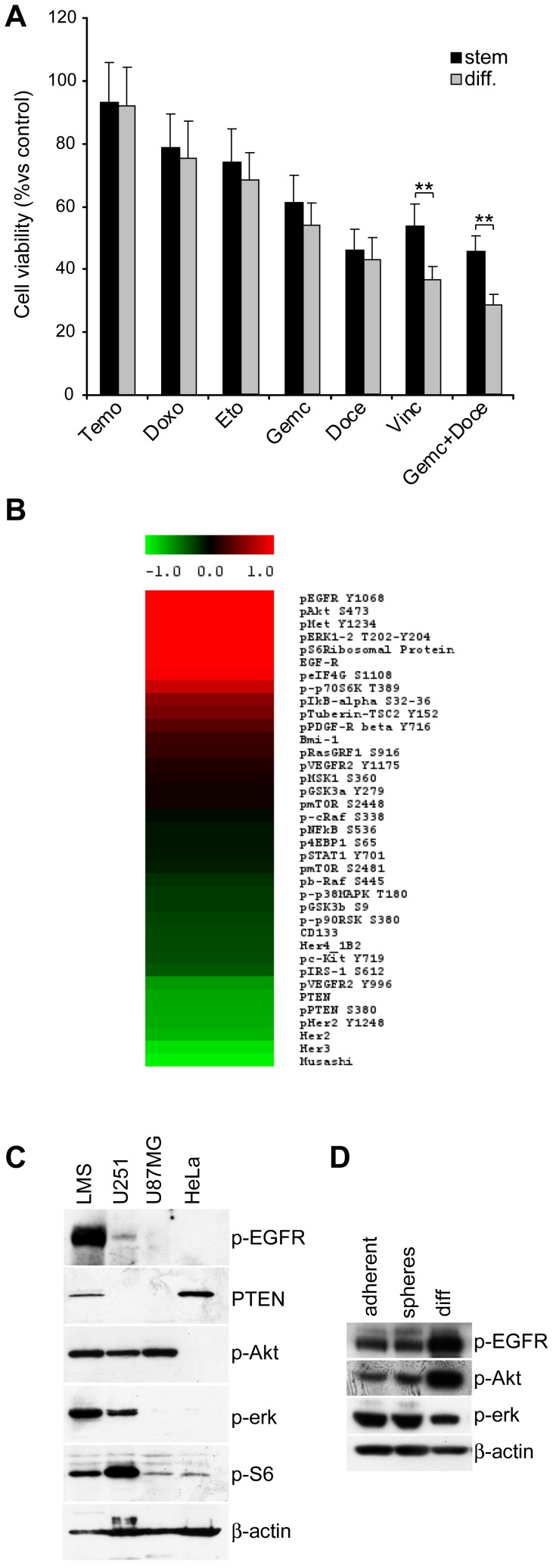
LMS stem-like cells are chemoresistant and display high activation of Akt and Erk pathways. A) Cell viability (% vs control, measured by cell titer glo luminescence) of undifferentiated and differentiated LMS cells exposed for 3 days to the indicated drugs. Mean ± SD of 3 independent experiments is shown. ** p<0,01. B) Reverse Phase Phosphoproteomic Array (RPPA) of LMS CSC. Standardized levels of expression or phosphorylation of the indicated proteins are reported. Standardized values were calculated for each antibody by subtracting the mean and dividing by the standard deviation of the sample series. Color scale limits from red to green include values spanning from ≥1,5 to ≤−1,5 standard deviations. C) Immunoblot analysis for the indicated proteins in LMS stem-like or control (U251, U87MG, HeLa) cells. D) Immunoblot analysis for the indicated proteins in LMS-stem like (adherent cultures or spheres) or differentiated (diff) cells.

The ability to reproduce patient tumors in immunocompromised animals may provide key information on the potential efficacy of drugs or drug-combinations at the CSC level. Therefore, we evaluated the cytotoxic activity of the currently used antineoplastic agents on LMS stem-like cells. In order to mimic patient regimens, LMS-CSC were exposed to gemcitabine, vincristine, doxorubicin, temozolomide, docetaxel, etoposide, and dacarbazine, as single agents or to the gemcitabine/docetaxel combination at doses comparable to those reached in treated patients before measuring cell viability. In line with the scarce patient response and frequent patient relapse to treatments, LMS stem-like cells resulted resistant or only slightly sensitive to most compounds tested (Figure 4). Some drugs, such as vincristine, gemcitabine and docetaxel displayed a considerable activity against LMS stem-like cells, whereas temozolomide, doxorubicin and etoposide resulted slightly effective (Figure 4A). Although drugs were partially cytotoxic, none of the tested compounds significantly killed off LMS stem-like cells, confirming the need of alternative antineoplastic strategies to treat this tumor more effectively. In parallel, the differentiated LMS cells displayed a similar response to chemotherapy with a slightly increased sensitivity only to some chemotherapeutic agents, as vincristine and the docetaxel/gemcitabine combination (Figure 4A).

### Analysis of Genetic Alterations and Pathway Activation of LMS Stem-like Cells

In order to find potentially exploitable therapeutic targets for killing LMS stem-like cells, we investigated the activation of several survival/oncogenic pathways by the Reverse Phase Phosphoproteomic Array (RPPA) evaluating the expression levels of a platform of selected key proteins and phosphoproteins. We found a strong activation of the EGFR and downstream pathways Akt and MAPK (Figure 4B). These results were confirmed by immunoblot analysis of LMS stem-like cells in comparison with control cell lines. We found high levels of phosphorylated EGFR, Akt, Erk, S6 and low levels of PTEN (Figure 4C). Thus, increase in EGFR phosphorylation and decreased expression of PTEN may contribute to the activation of Akt and Erk pathways, suggesting the possibility to overcome LMS stem-like cell survival through the inhibition of EGFR. Differentiated LMS cells displayed even higher EGFR pathway activation than undifferentiated cells suggesting that EGFR could represent a potential candidate for targeting both stem and differentiated LMS cells (Figure 4D). Both sarcospheres and adherent undifferentiated LMS cells displayed similar levels of p-EGFR, p-Akt and p-Erk, confirming the assumption that LMS stem-like cells obtained with the two alternative approaches do not differ significantly (Figure 4D). Accordingly, we investigated by DNA sequence the presence of genomic alterations in genes that may be linked to these altered pathways and contribute to the tumor chemoresistance. As reported in Table S1, EGFR, Akt, PTEN, PI3K genes resulted not mutated, suggesting different mechanisms leading to pathway activation.

### EGFR Inhibition Leads to Reduction of Akt and Erk Pathway Activation and LMS Stem-like Cell Chemo-sensitization *in vitro*

**Figure 5:**
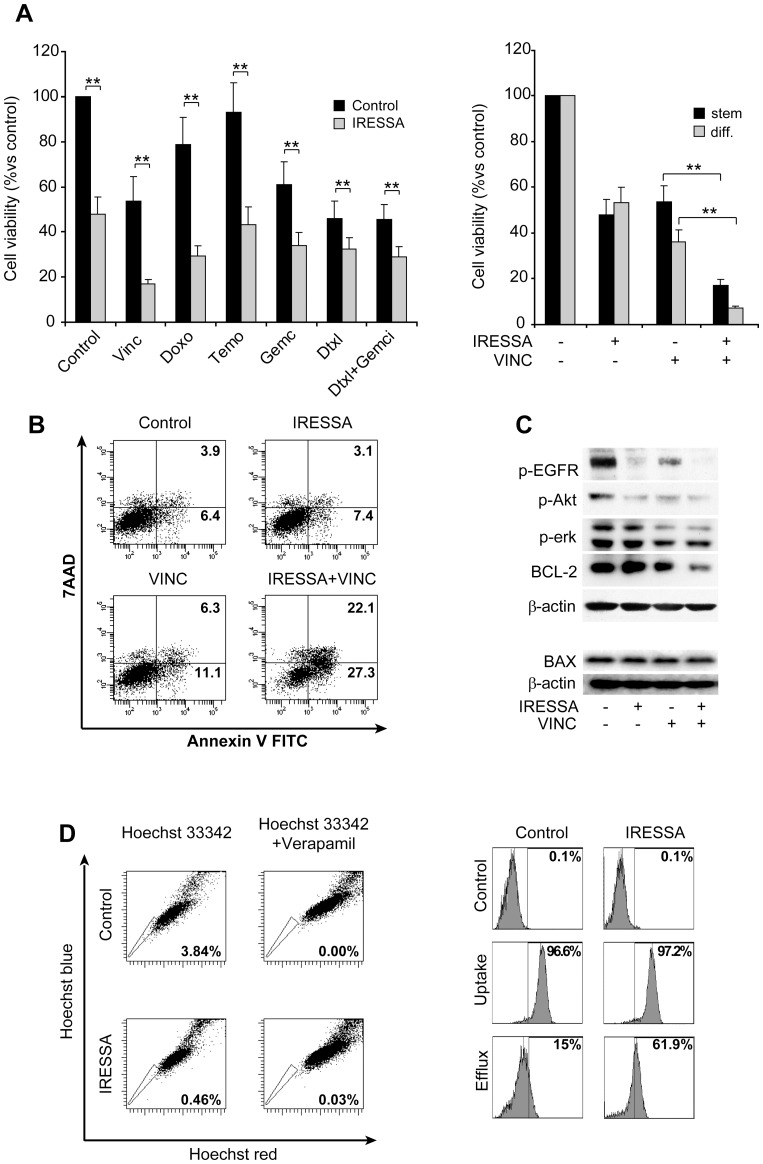
IRESSA treatment reduces LMS stem-like cell growth rate and sensitizes them to chemotherapy. A) *Effect of IRESSA on LMS cell chemo-sensitivity*. (Left) Stem-like cells were exposed to IRESSA and the indicated compounds and cell viability was evaluated after 72 hours by Cell Titer Glo assay (Promega). (Right) Cell viability of undifferentiated (stem) or differentiated (diff) LMS cells exposed to IRESSA/vincristine combination for 72 hours. Mean ± SD of 3 independent experiments is shown. ** p<0,01. B) Flow cytometric analysis for Annexin V of LMS stem-like cells untreated (control) or treated as indicated. C) Immunoblot for the indicated proteins or phosphoproteins of LMS stem-like cells left untreated or treated as indicated. D) *Effect of IRESSA on SP and drug efflux ability.* (Left) SP analysis of control or IRESSA-treated LMS stem-like cells. (Right) Cytoﬂuorimetric profiles of control or IRESSA treated LMS stem-like cells after exposure to doxorubicin (Uptake), or after drug treatment followed by removal and overnight incubation in drug-free medium (Efﬂux).

The active state of Akt and Erk pathways suggested the possibility to abolish or reduce LMS stem-like cell chemoresistance through targeting such survival signals. Inhibition of EGFR could represent a promising strategy to achieve concomitant down-regulation of both downstream signaling pathways possibly leading to LMS stem-like cell killing, growth inhibition or chemosensitization. Therefore, we investigated LMS stem-like cell response to the EGFR inhibitor IRESSA (Gefitinib) used as single agent or in combination with chemotherapy. When used as single agent, IRESSA reduced cell growth in the absence of cytotoxicity, as determined by cell inspection, which revealed negligible amounts of dead cells both in control or IRESSA-treated samples (Figure S3A and data not shown). The combined use of IRESSA and chemotherapeutic drugs resulted in additive anti-tumor effect, with the highest cell death rate obtained in combination with vincristine, both on LMS stem and differentiated cells (Figure 5A, 5B and Figure S2B). Cell cycle analysis showed that such combination further increased the G2/M cell fraction (Figure S2C), confirming that treatment with IRESSA potentiates the activity of vincristine. Cell death occurred mainly through apoptosis as suggested by morphological features and annexin V binding of treated cells (Figure S2B and Figure 5B). Reduced phosphorylation levels of EGFR, Akt and Erk in vincristine/IRESSA-treated cells indicated the inhibition of the whole pathway downstream EGFR (Figure 5C). Furthermore, the combined treatment determined a strong reduction in the anti-apoptotic factor Bcl-2 levels, confirming that EGFR inhibition potentiates vincristine effects also in terms of Bcl-2 down-modulation (Figure 5C) [36]. In contrast, the levels of the anti-apoptotic protein BAX did not vary with treatment, indicating that the observed Bcl-2 decrease is a specific effect of treatment. The considerable *in vitro* cytotoxic activity of vincristine in combination with IRESSA may not be entirely explained by the inhibition of survival pathways. The ability of LMS stem-like cells to extrude cytotoxic drugs may contribute to their relative chemoresistance. Given that IRESSA has been shown to inhibit membrane pump activity and favor drug retention within tumors [37], [38], we investigated whether a similar effect could be obtained in LMS stem-like cells, ultimately contributing to their death. The analysis of Hoechst 33342 retention showed that IRESSA exposure determined a marked reduction of the SP fraction within the stem-like cell population (Figure 5D left), in parallel with a prolonged accumulation of doxorubicin (Figure 5D right). These results suggest that IRESSA enhances the cytotoxic activity of chemotherapeutic agents by both targeting survival pathways downstream EGFR and increasing drug retention.

### IRESSA/Vincristine Treatment Results in Strong Anti-tumor Activity in Patient Tumor Phospho-copies Generated by LMS Stem-like Cells

**Figure 6:**
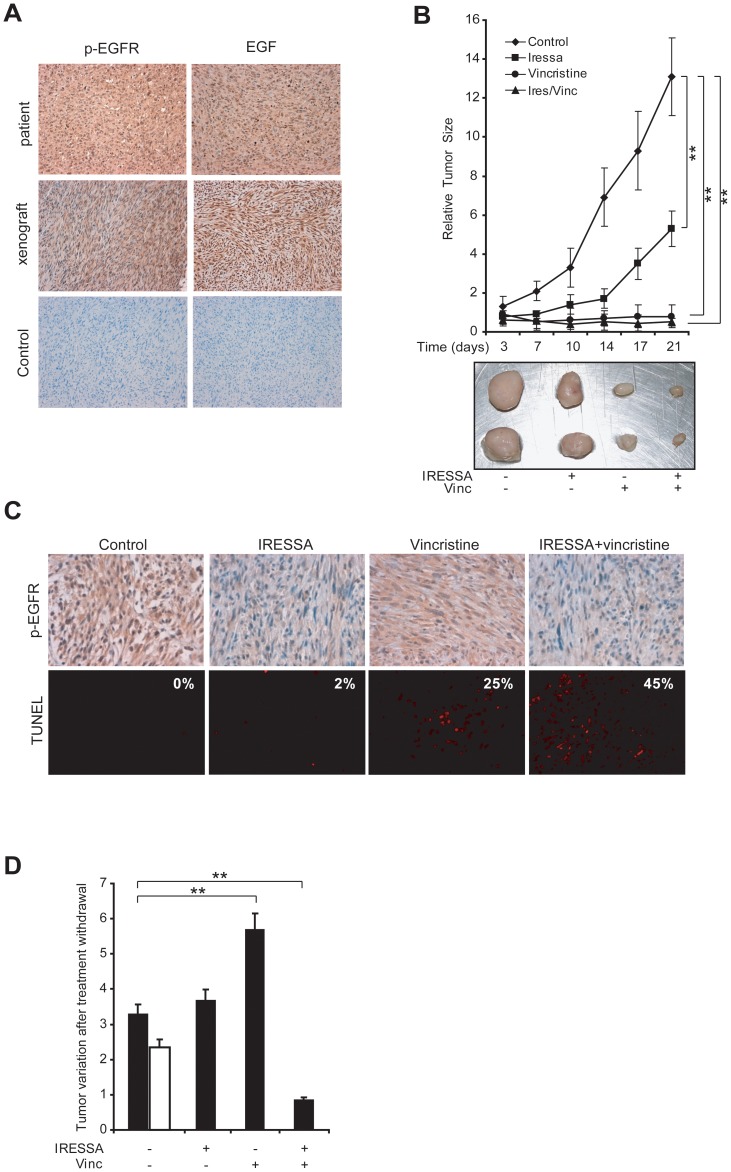
IRESSA/Vincristine treatment results in strong stem-like cell-effective anti-tumor activity in patient tumor phospho-copies generated by LMS-stem-like cells. A) LMS stem-like cells derived xenografts display high levels of EGF and p-EGFR similarly to the patient tumor. A) EGF and p-EGFR immunohistochemistry in patient tumor specimens and in LMS-CSC derived xenografts. B) Tumor growth curves and tumor pictures of LMS-CSC derived xenografts in control, IRESSA-, Vincristine- or IRESSA+Vincristine- treated mice. Mean ± SD of 3 independent experiments is shown. **p<0,01. C) p-EGFR immunohistochemistry and TUNEL assay of control or treated xenografts. The percentage of Tunel positive cells is indicated for each condition. D) Tumor growth rate of control or pre-treated tumors after treatment interruption. Pre-treated tumors were monitored after 10 days and tumor growth is indicated as ratio of tumor volume at day10 vs day0.The white bar represents growth rate of additional control tumors comparable to treated tumors in size. Mean ± SD of 3 independent experiments is shown. ** p<0,01.

To rule out the possibility that the EGFR activation observed in LMS stem-like cells could result from culture-induced modifications, we investigated the activation state of the EGFR pathway in the original patient tumor. In the absence of EGFR mutations in tumor cells (see Table S1), we hypothesized that receptor stimulation might depend on the production of high EGF levels in the tumor microenvironment. The immunohistochemical analysis showed the presence of a significant amount of EGF in the parental LMS, together with diffuse phosphorylation of EGFR (Figure 6A). Of note, a similar pattern of EGF and p-EGFR expression was observed in LMS stem-like cell-based tumor xenografts (Figure 6A), confirming that this experimental model may provide a suitable tool for preclinical testing of EGFR inhibition.

NOD-SCID mice bearing subcutaneous LMS stem-like cell-generated tumors were treated with Vincristine, IRESSA, or their combination. Similarly to *in vitro* results, treatment with IRESSA reduced tumor growth, whereas vincristine displayed more striking antitumor activity, particularly in combination with IRESSA (Figure 6 B), as shown also by the macroscopic appearance of excised tumors and tumor weights at the end of treatment (Image in Figure 6B and results not shown). At the end of treatment most tumors were collected and analyzed for *in vivo* EGFR inhibition and apoptosis induction. The immunohistochemistry analysis showed reduced levels of pEGFR in treated samples when compared to controls (6C top panels), indicating that the IRESSA levels reached *in vivo* were sufficient to achieve EGFR inhibition and confirming that the antitumor effect of IRESSA was mediated by inhibition of EGFR. In addition, TUNEL assay revealed a consistent fraction of apoptotic cells in vincristine and, to a higher extent, in double treated-tumors, while control and IRESSA treated tumors did not show any sign of apoptotic cell death (Figure 6C bottom panels).

In order to evaluate whether the single or combined treatments were able to affect the tumorigenic cell population *in vivo,* we monitored the tumor size after treatment withdrawal. Tumors were monitored for 10 days with the aim of investigating the ability of the single or combined treatments to abolish or delay tumor re-growth. We observed that after therapy cessation, tumors from vincristine-treated mice started to re-grow, whereas those subjected to combined treatment continued to decrease in size and recurred only after several weeks (Figure 6D and data not shown). This effect was not due to the different size of treated and untreated tumors, since we obtained similar results when we compared the growth rate of treated tumors with controls of similar volumes (Figure 6D). Finally, in order to investigate whether EGF/EGFR pathway activation was a common signature in LMS, we analyzed a panel of 10 LMS patient-derived specimens. All samples analyzed revealed high or moderate expression of EGF and p-EGFR levels (Figure S4A). The expression was diffuse in all cells of the tissue although the intensity varied in different samples, having always higher levels than in normal samples (Figure S4B). These results indicated that the EGF/EGFR pathway is frequently activated in LMS.

## Discussion

LMS patients often relapse following surgical and radiation treatment. Although some patients respond to the current second-line chemotherapeutic regimens, the prognosis remain particularly poor.

Many reports suggest that CSC have a pivotal role in tumor chemoresistance and relapse. The ability to expand *in vitro* leiomyosarcoma stem-like cells represent a powerful tool to obtain their comprehensive characterization, which may represent the first step toward the development of effective treatments.

Here, we report for the first time the expansion of leiomyosarcoma initiating cells and investigated the mechanisms underlying their weak response to most of the current treatments as well as the possibility to increase the antitumor effect of standard chemotherapy by concurrent inhibition of the survival pathways activated in the LMS stem-like cells.

Immature LMS cells could be obtained by standard serum-free culture conditions that allow selection and expansion of immature tumor cells as “spheres”, as we previously showed for CSC of various solid tumors [18], [19], [21]. Additionally, based on the assumption that LMS-CSC may be considered as transformed MSC, we were able to efficiently expand the LMS-CSC in culture conditions widely used for non transformed MSC of various origin, thus identifying an additional method for obtaining stem-like cell-enriched cultures from mesenchymal tumors. Alternatively, another possibility might be that, rather than the expansion of undifferentiated cells, the stem cell suitable culture conditions, different from standard culture conditions, might influence the antigenic pattern of cultured cells toward a MCS- like phenotype. However, we found that both types of stem cell-suitable cultures, although completely different, generated cultures with similar antigenic pattern, suggesting the possibility of a function-based selection of cells rather than phenotype remodeling.

*In vitro* expanded LMS stem-like cells were endowed with stem-like cell potential *in vitro* and high tumorigenic potential coupled with the ability to generate primary and secondary patient-like tumors in immunodeficient mice, indicating that such *in vitro* expanded LMS stem-like cells were able to recapitulate the tumorigenic process by proliferating *in vivo* while both self-renewing and producing a progeny of differentiated cells that constitute the major cellular population in the tumor. In comparison to differentiated cells, stem-like cells displayed increased tumorigenicity not only in terms of reduced latency for tumor growth but also in terms of tumor formation efficiency. In particular, low numbers of differentiated cells (10^4^) displayed a highly reduced ability to generate tumors even after long periods of observation while stem-like cells displayed about 100% efficency when the same number of cells were injected.

Of note, the same experimental strategies proved to be effective in the *in vitro* long term expansion of undifferentiated cells from other sarcomas (results not shown). Such *in vitro* expanded LMS stem-like cells displayed the surface antigen expression expected for undifferentiated tumor cells of mesenchymal origin.

*In vitro* expanded LMS stem-like cells contained a significant fraction of cells with SP phenotype which showed a slightly increased tumorigenic activity in comparison to the whole population of undifferentiated cells (Figure S5).

Long term expansion of LMS stem-like cells allowed their extensive investigation, including the evaluation of their chemosensitivity. Exposure of LMS- stem-like cells to anti-neoplastic agents currently used in the clinical practice or in clinical trials resulted in a modest cytotoxicity. Concerning chemosensitivity, although both stem and differentiated LMS cells resulted quite resistant to most treatments, some drugs (in particular vincristine and the combination docetaxel/gemcitabine) displayed higher cytotoxicity against differentiated cells. In addition, it is important to mention that, even if chemoresistance is not limited to CSCs, targeting this tumorigenic population appears to be required to obtain long-term antitumor effect. Thus, even a similar degree of chemoresistance observed in our experimental conditions in the two types of cells may be more relevant when associated to CSC than to differentiated cells, the latter characterized by limited life span.

In the absence of genetic alterations in the Akt, PI3K, PTEN and EGFR genes (Table S1), we investigated the activation status of key survival pathways possibly contributing to chemoresistance. EGFR and key proteins belonging to the downstream PI3/AKT and MAPK/ERK pathways were considerably activated in LMS stem like cells, while the treatment with IRESSA resulted in inhibition of the EGFR, AKT and ERK pathway, with consequent sensitization to several chemotherapeutic agents.

We found that EGFR inhibition by IRESSA determined a marked reduction of the drug efflux activity and of the SP fraction within the LMS stem-like cells. Thus, the strong chemo-sensitization effect of IRESSA may rely on its ability to affect both survival pathway signaling and active drug extrusion.

LMS stem-like cells were able to generate patient-like tumors also in terms of EGFR activation, a prerequisite for preclinical testing of antitumor compounds targeting this receptor. IRESSA plus Vincristine treatments were highly cytotoxic and were found to strongly counteract tumor growth. However, vincristine-treated tumors immediately started to re-grow after treatment cessation. Their growth rate even increased compared to non-pretreated tumors, while double treatment caused a long delay before the tumor started to slowly re-grow. A plausible interpretation of these results may be that the relative number of tumorigenic cells could be enriched by vincristine treatment and not by combined therapy. In other words vincristine most likely affected viability of differentiated tumor cells sparing LMS-stem-like cells, while the combination treatment was effective against both stem-like and differentiated cells. This is in line with recent data from our group showing that in different solid tumors chemotherapy spares quiescent rather than proliferating cancer stem-like cells [39], [40]. However, another interpretation might be that vincristine could determine the selection of cells with increased proliferative potential. What is not questionable is that tumors treated with chemotherapy were growing more actively after interrupting treatment while IRESSA-chemo treated tumors did not regrow for a longer period of time. Therefore, it is reasonable to hypothesize that similarly to mice such CSC-effective therapeutic strategy might determine a longer progression free survival in patients and a slow progression of relapsing tumors.

Previous studies showed that EGFR targeting combined with chemotherapy displayed marked antitumor activity against some sarcoma cell lines both *in vitro* and *in vivo* [41], [42]. Our results are in agreement with these studies and further point toward this treatment direction demonstrating for the first time the efficacy of EGFR targeting in chemosensitization of LMS stem-like cells. Furthermore, finding that IRESSA, besides EGFR pathway inactivation, strongly inhibits the stem cell-associated active drug extrusion, may explain the efficacy of the combination therapy against the tumorigenic cells. The detection of EGFR phosphorylation in all the LMS analyzed suggests that EGFR targeting agents may constitute a valuable therapeutic tool for those LMS patients lacking driving molecular alteration downstream of EGFR. High levels of EGF within the tumor may possibly be responsible for receptor activation and suggest the possibility to obtain anticancer activity against LMS through the direct inhibition of EGF. However, it remains to be elucidated whether EGF is secreted by cancer or stroma cells.

In conclusion, our study proposed a potential therapeutic strategy that might be effective for LMS patients with EGFR pathway activation. The efficacy of this therapeutic strategy most likely relies on its multitarget activity, as it resulted both in a simultaneous inactivation of multiple oncogenic signals, and in the inhibition of CSC-linked properties leading to cytotoxicity against the more aggressive tumorigenic cell population. Additional functional studies will extend our analysis to larger numbers of LMS patient tumors, aiming to increase reliability of results and provide potential alternative treatments to lengthen survival of relapsed LMS patients unresponsive to current clinical regimens.

## Supporting Information

Figure S1 A) Tumorigenicity of stem and differentated LMS cells. Percentage of tumor positive mice after injection of low numbers of stem-like or differentiated LMS cells. 5 mice were injected with 10^4^ cells and tumor formation evaluated after 4 months. B) *In vivo differentiation of LMS stem-like cells*. Flow cytometry analysis for CD105 and CD146 expression in parental tumor, sarcospheres (S), adherent culture (A) or differentiated LMS cells obtained from freshly dissociated xenografts generated by sarcosphere or adherent culture cell injection. The percentage of positive cells is represented by the different histograms corresponding to the cell populations or tumor types as indicated.
(TIF)

Figure S2 A) Immunohistochemistry for the indicated antigens performed on patient tumor (left), tumor generated by subcutaneous injection of LMS spheres (middle) or adherent undifferentiated cells (right). B) Tumor growth rate of undifferentiated LMS cells (adherent cultures) injected subcutaneously in NOD-SCID mice with or without Matrigel as indicated. The values represent mean +/− SD of three independent experiments. Student’ s T test was used to determine p-value. ***p<0,001. C) Hematoxylin and eosin (H&E) or immunohistochemistry for the indicated antigens performed on secondary tumors or tumors generated by differentiated LMS cells, as indicated.
(TIF)

Figure S3 A) Effect of IRESSA on LMS stem-like cell proliferation. LMS stem like cells were plated and left untreated or exposed to IRESSA for the indicated time points. Cell growth is indicated as percentage of treated cell versus control cell numbers ateach time. B) Morphologycal appearance of LMS stem-like cells untreated (control) or treated 3 days as indicated. C) Cell cycle distribution of the same cells as in B after 2 days drug esposure.
(TIF)

Figure S4 EGF/EGFR pathway is generally activated in leiomyosarcomas. A) p-EGFR and EGF immunohistochemistry in 3 out of 10 representative patient-derived LMS specimens. B) *Table showing the EGF and pEGFR expression in 10* LMS patient-derived specimens and in 5 non tumoral tissue specimen (Myometrium). Values 1 to 4 indicating the percentage of positive cells with 0 = negative, 1<10%; 2 = 10–25%; 3 = 25–50%, 4<50% and letters A to C indicating the intensity of expression (A = weak,; B = moderate; C = high intensity).
(TIF)

Figure S5 A) Cytofluorimetric cell sorting of side population (SP) cells (right panel). B) Tumor growth rate of xenografts generated by subcutaneous injection of sorted SP cells and unsorted undifferentiated LMS cells (adherent cultures). The values represent mean +/− SD of three independent experiments. Student’ s T test was used to determine p-value. **p<0,01.
(TIF)

Table S1 Genetic pattern of LMS stem-like cells. The specific primers used for amplification of the listed genes are reported. PCR products were analyzed and compared with the corresponding Genebank sequences of each gene for the presence of tumor-associated alterations. The status of DNA is indicated as wt when similarity among PCR product and genebank sequence was 100%.
(XLS)

## References

[1] Jemal A, Siegel R, Ward E, Hao Y, Xu J, et al (2008) Cancer statistics, 2008. CA Cancer J Clin 58: 71–96. 18287387 10.3322/CA.2007.0010

[2] Mackall CL, Meltzer PS, Helman LJ (2002) Focus on sarcomas. Cancer Cell 2: 175–178. 12242149 10.1016/s1535-6108(02)00132-0

[3] Osuna D, de Alava E (2009) Molecular pathology of sarcomas. Rev Recent Clin Trials 4: 12–26. 19149759 10.2174/157488709787047585

[4] Rubio R, Garcia-Castro J, Gutierrez-Aranda I, Paramio J, Santos M, et al (2010) Deficiency in p53 but not retinoblastoma induces the transformation of mesenchymal stem cells in vitro and initiates leiomyosarcoma in vivo. Cancer Res 70: 4185–4194. 20442289 10.1158/0008-5472.CAN-09-4640

[5] Rodriguez R, Rubio R, Menendez P (2012) Modeling sarcomagenesis using multipotent mesenchymal stem cells. Cell Res 22: 62–77. 21931359 10.1038/cr.2011.157 PMC3351912

[6] Weitz J, Antonescu CR, Brennan MF (2003) Localized extremity soft tissue sarcoma: improved knowledge with unchanged survival over time. J Clin Oncol 21: 2719–2725. 12860950 10.1200/JCO.2003.02.026

[7] Coindre JM, Terrier P, Guillou L, Le Doussal V, Collin F, et al (2001) Predictive value of grade for metastasis development in the main histologic types of adult soft tissue sarcomas: a study of 1240 patients from the French Federation of Cancer Centers Sarcoma Group. Cancer 91: 1914–1926. 11346874 10.1002/1097-0142(20010515)91:10<1914::aid-cncr1214>3.0.co;2-3

[8] Penel N, Italiano A, Isambert N, Bompas E, Bousquet G, et al (2009) Factors affecting the outcome of patients with metastatic leiomyosarcoma treated with doxorubicin-containing chemotherapy. Ann Oncol 21(6): 1361–1365. 19880438 10.1093/annonc/mdp485

[9] Van Glabbeke M, van Oosterom AT, Oosterhuis JW, Mouridsen H, Crowther D, et al (1999) Prognostic factors for the outcome of chemotherapy in advanced soft tissue sarcoma: an analysis of 2,185 patients treated with anthracycline-containing first-line regimens–a European Organization for Research and Treatment of Cancer Soft Tissue and Bone Sarcoma Group Study. J Clin Oncol 17: 150–157. 10458228 10.1200/JCO.1999.17.1.150

[10] Blay JY, van Glabbeke M, Verweij J, van Oosterom AT, Le Cesne A, et al (2003) Advanced soft-tissue sarcoma: a disease that is potentially curable for a subset of patients treated with chemotherapy. Eur J Cancer 39: 64–69. 12504660 10.1016/s0959-8049(02)00480-x

[11] Krikelis D, Judson I (2010) Role of chemotherapy in the management of soft tissue sarcomas. Expert Rev Anticancer Ther 10: 249–260. 20132000 10.1586/era.09.176

[12] Hensley ML, Maki R, Venkatraman E, Geller G, Lovegren M, et al (2002) Gemcitabine and docetaxel in patients with unresectable leiomyosarcoma: results of a phase II trial. J Clin Oncol 20: 2824–2831. 12065559 10.1200/JCO.2002.11.050

[13] Talbot SM, Keohan ML, Hesdorffer M, Orrico R, Bagiella E, et al (2003) A phase II trial of temozolomide in patients with unresectable or metastatic soft tissue sarcoma. Cancer 98: 1942–1946. 14584078 10.1002/cncr.11730

[14] Garcia-Carbonero R, Supko JG, Manola J, Seiden MV, Harmon D, et al (2004) Phase II and pharmacokinetic study of ecteinascidin 743 in patients with progressive sarcomas of soft tissues refractory to chemotherapy. J Clin Oncol 22: 1480–1490. 15084621 10.1200/JCO.2004.02.098

[15] Hernando E, Charytonowicz E, Dudas ME, Menendez S, Matushansky I, et al (2007) The AKT-mTOR pathway plays a critical role in the development of leiomyosarcomas. Nat Med 13: 748–753. 17496901 10.1038/nm1560

[16] Oda Y, Saito T, Tateishi N, Ohishi Y, Tamiya S, et al (2005) ATP-binding cassette superfamily transporter gene expression in human soft tissue sarcomas. Int J Cancer 114: 854–862. 15609299 10.1002/ijc.20589

[17] Visvader JE, Lindeman GJ (2008) Cancer stem cells in solid tumours: accumulating evidence and unresolved questions. Nat Rev Cancer 8: 755–768. 18784658 10.1038/nrc2499

[18] Eramo A, Lotti F, Sette G, Pilozzi E, Biffoni M, et al (2008) Identification and expansion of the tumorigenic lung cancer stem cell population. Cell Death Differ 15: 504–514. 18049477 10.1038/sj.cdd.4402283

[19] Eramo A, Ricci-Vitiani L, Zeuner A, Pallini R, Lotti F, et al (2006) Chemotherapy resistance of glioblastoma stem cells. Cell Death Differ 13: 1238–1241. 16456578 10.1038/sj.cdd.4401872

[20] O’Brien CA, Pollett A, Gallinger S, Dick JE (2007) A human colon cancer cell capable of initiating tumour growth in immunodeficient mice. Nature 445: 106–110. 17122772 10.1038/nature05372

[21] Ricci-Vitiani L, Lombardi DG, Pilozzi E, Biffoni M, Todaro M, et al (2007) Identification and expansion of human colon-cancer-initiating cells. Nature 445: 111–115. 17122771 10.1038/nature05384

[22] Zhou BB, Zhang H, Damelin M, Geles KG, Grindley JC, et al (2009) Tumour-initiating cells: challenges and opportunities for anticancer drug discovery. Nat Rev Drug Discov 8: 806–823. 19794444 10.1038/nrd2137

[23] Singh SK, Hawkins C, Clarke ID, Squire JA, Bayani J, et al (2004) Identification of human brain tumour initiating cells. Nature 432: 396–401. 15549107 10.1038/nature03128

[24] Al-Hajj M, Wicha MS, Benito-Hernandez A, Morrison SJ, Clarke MF (2003) Prospective identification of tumorigenic breast cancer cells. Proc Natl Acad Sci U S A 100: 3983–3988. 12629218 10.1073/pnas.0530291100 PMC153034

[25] Levings PP, McGarry SV, Currie TP, Nickerson DM, McClellan S, et al (2009) Expression of an exogenous human Oct-4 promoter identifies tumor-initiating cells in osteosarcoma. Cancer Res 69: 5648–5655. 19584295 10.1158/0008-5472.CAN-08-3580 PMC2841219

[26] Gibbs CP, Kukekov VG, Reith JD, Tchigrinova O, Suslov ON, et al (2005) Stem-like cells in bone sarcomas: implications for tumorigenesis. Neoplasia 7: 967–976. 16331882 10.1593/neo.05394 PMC1502023

[27] Tirino V, Desiderio V, d’Aquino R, De Francesco F, Pirozzi G, et al (2008) Detection and characterization of CD133+ cancer stem cells in human solid tumours. PLoS One 3: e3469. 18941626 10.1371/journal.pone.0003469 PMC2565108

[28] Wu C, Wei Q, Utomo V, Nadesan P, Whetstone H, et al (2007) Side population cells isolated from mesenchymal neoplasms have tumor initiating potential. Cancer Res 67: 8216–8222. 17804735 10.1158/0008-5472.CAN-07-0999

[29] Honoki K (2010) Do stem-like cells play a role in drug resistance of sarcomas? Expert Rev Anticancer Ther 10: 261–270. 20132001 10.1586/era.09.184

[30] Pierobon M, Calvert V, Belluco C, Garaci E, Deng J, et al (2009) Multiplexed Cell Signaling Analysis of Metastatic and Nonmetastatic Colorectal Cancer Reveals COX2-EGFR Signaling Activation as a Potential Prognostic Pathway Biomarker. Clin Colorectal Cancer 8: 110–117. 10.3816/CCC.2009.n.018 19423505

[31] Sorrentino A, Ferracin M, Castelli G, Biffoni M, Tomaselli G, et al (2008) Isolation and characterization of CD146+ multipotent mesenchymal stromal cells. Exp Hematol 36: 1035–1046. 18504067 10.1016/j.exphem.2008.03.004

[32] Mohseny AB, Hogendoorn PC (2011) Mesenchymal Tumors: When Stem Cells Go Mad. Stem Cells. 29(3): 397–403. 10.1002/stem.596 21425403

[33] Valtieri M, Sorrentino A (2008) The mesenchymal stromal cell contribution to homeostasis. J Cell Physiol 217: 296–300. 18615579 10.1002/jcp.21521

[34] Halfon S, Abramov N, Grinblat B, Ginis I (2011) Markers distinguishing mesenchymal stem cells from fibroblasts are downregulated with passaging. Stem Cells Dev 20: 53–66. 20528146 10.1089/scd.2010.0040

[35] Romanov YA, Svintsitskaya VA, Smirnov VN (2003) Searching for alternative sources of postnatal human mesenchymal stem cells: candidate MSC-like cells from umbilical cord. Stem Cells 21: 105–110. 12529557 10.1634/stemcells.21-1-105

[36] Thomadaki H, Floros KV, Scorilas A (2009) Molecular response of HL-60 cells to mitotic inhibitors vincristine and taxol visualized with apoptosis-related gene expressions, including the new member BCL2L12. Ann N Y Acad Sci 1171: 276–283. 19723066 10.1111/j.1749-6632.2009.04912.x

[37] Noguchi K, Kawahara H, Kaji A, Katayama K, Mitsuhashi J, et al (2009) Substrate-dependent bidirectional modulation of P-glycoprotein-mediated drug resistance by erlotinib. Cancer Sci 100: 1701–1707. 19493273 10.1111/j.1349-7006.2009.01213.x PMC11159693

[38] Carcaboso AM, Elmeliegy MA, Shen J, Juel SJ, Zhang ZM, et al (2010) Tyrosine kinase inhibitor gefitinib enhances topotecan penetration of gliomas. Cancer Res 70: 4499–4508. 20460504 10.1158/0008-5472.CAN-09-4264 PMC2880208

[39] Bartucci M, Svensson S, Romania P, Dattilo R, Patrizii M, et al (2012) Therapeutic targeting of Chk1 in NSCLC stem cells during chemotherapy. Cell Death Differ 19: 768–778. 22117197 10.1038/cdd.2011.170 PMC3321626

[40] Francescangeli F, Patrizii M, Signore M, Federici G, Di Franco S, et al (2012) Proliferation State and Polo-like Kinase1 Dependence of Tumorigenic Colon Cancer Cells. Stem Cells 30(9): 1819–1830. 22753241 10.1002/stem.1163

[41] Ren W, Korchin B, Zhu QS, Wei C, Dicker A, et al (2008) Epidermal growth factor receptor blockade in combination with conventional chemotherapy inhibits soft tissue sarcoma cell growth in vitro and in vivo. Clin Cancer Res 14: 2785–2795. 18451246 10.1158/1078-0432.CCR-07-4471

[42] Ren W, Korchin B, Lahat G, Wei C, Bolshakov S, et al (2008) Combined vascular endothelial growth factor receptor/epidermal growth factor receptor blockade with chemotherapy for treatment of local, uterine, and metastatic soft tissue sarcoma. Clin Cancer Res 14: 5466–5475. 18765538 10.1158/1078-0432.CCR-08-0562

