# Supplementary figures and images for: EGFR Inhibition Abrogates Leiomyosarcoma Cell Chemoresistance through Inactivation of Survival Pathways and Impairment of CSC Potential
Source: PLoS One. 2012 Oct 8;7(10):e46891. doi: 10.1371/journal.pone.0046891 (PMC3466184; doi:10.1371/journal.pone.0046891)

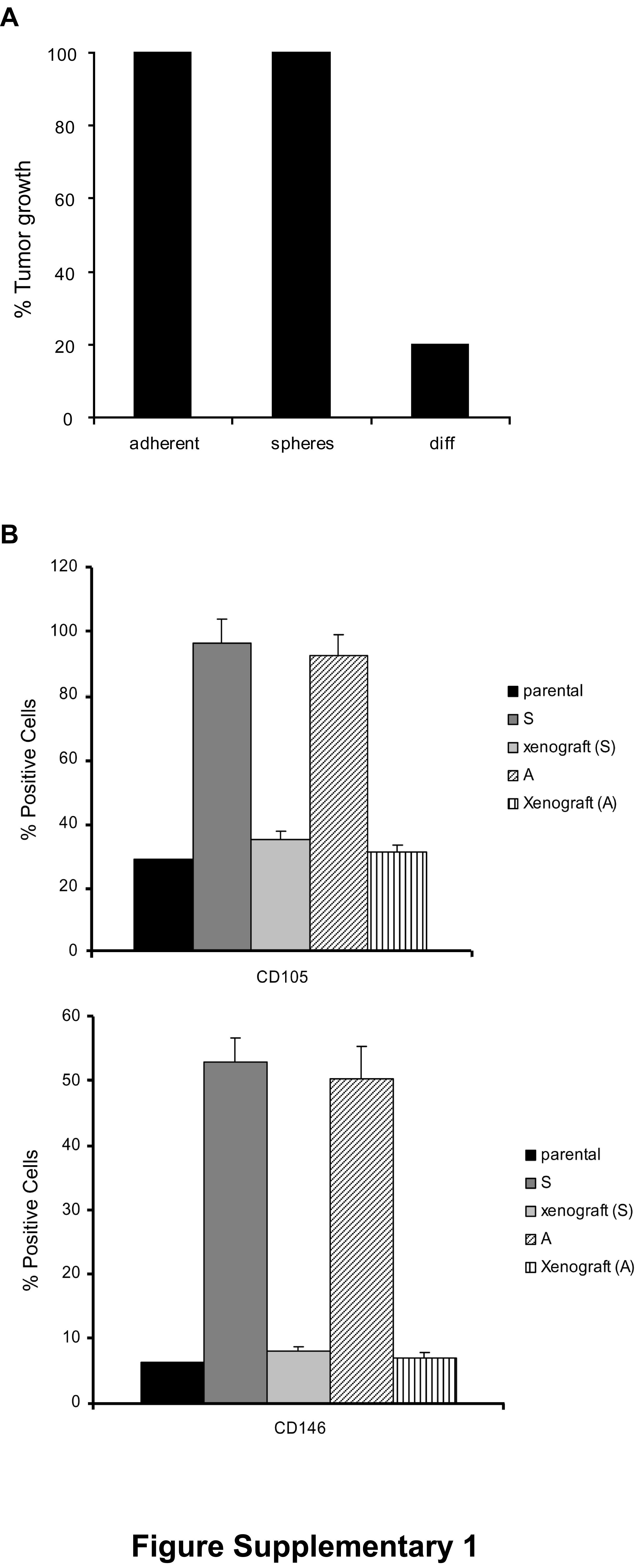

Supplement: Figure S1 — A) Tumorigenicity of stem and differentated LMS cells. Percentage of tumor positive mice after injection of low numbers of stem-like or differentiated LMS cells. 5 mice were injected with 104 cells and tumor formation evaluated after 4 months. B) In vivo differentiation of LMS stem-like cells. Flow cytometry analysis for CD105 and CD146 expression in parental tumor, sarcospheres (S), adherent culture (A) or differentiated LMS cells obtained from freshly dissociated xenografts generated by sarcosphere or adherent culture cell injection. The percentage of positive cells is represented by the different histograms corresponding to the cell populations or tumor types as indicated. (TIF) [file pone.0046891.s001.tif]

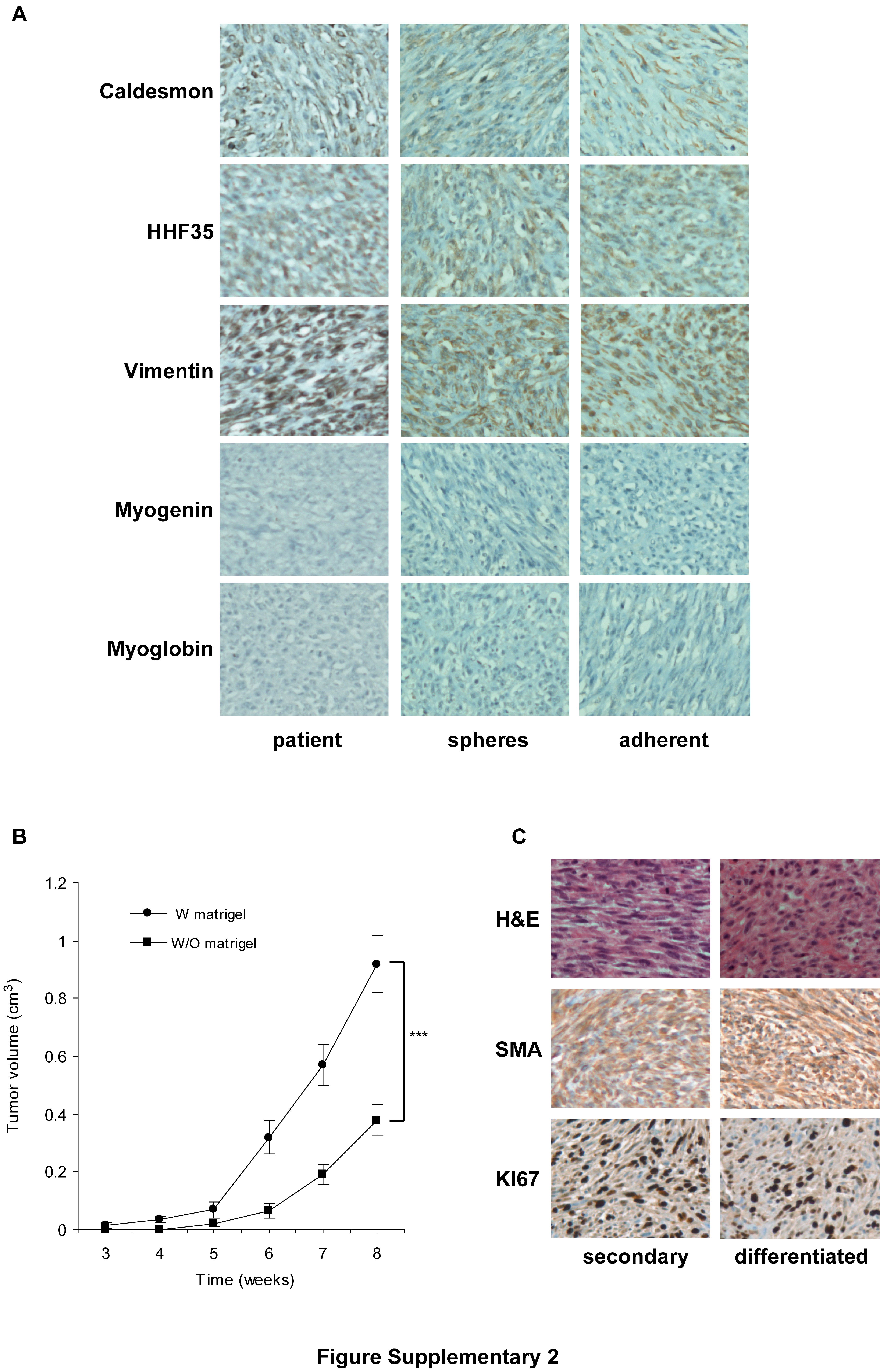

Supplement: Figure S2 — A) Immunohistochemistry for the indicated antigens performed on patient tumor (left), tumor generated by subcutaneous injection of LMS spheres (middle) or adherent undifferentiated cells (right). B) Tumor growth rate of undifferentiated LMS cells (adherent cultures) injected subcutaneously in NOD-SCID mice with or without Matrigel as indicated. The values represent mean +/− SD of three independent experiments. Student’ s T test was used to determine p-value. ***p<0,001. C) Hematoxylin and eosin (H&E) or immunohistochemistry for the indicated antigens performed on secondary tumors or tumors generated by differentiated LMS cells, as indicated. (TIF) [file pone.0046891.s002.tif]

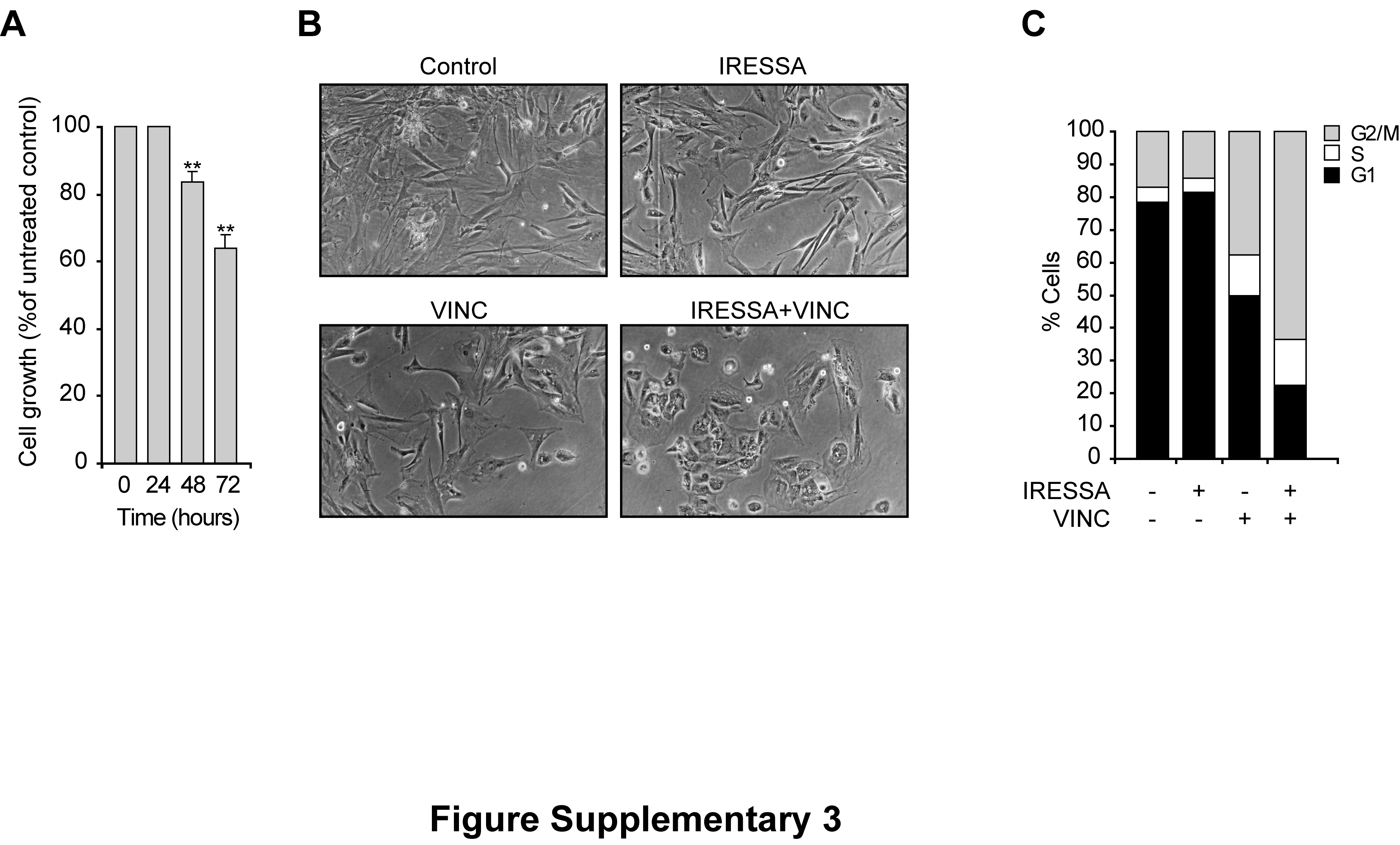

Supplement: Figure S3 — A) Effect of IRESSA on LMS stem-like cell proliferation. LMS stem like cells were plated and left untreated or exposed to IRESSA for the indicated time points. Cell growth is indicated as percentage of treated cell versus control cell numbers ateach time. B) Morphologycal appearance of LMS stem-like cells untreated (control) or treated 3 days as indicated. C) Cell cycle distribution of the same cells as in B after 2 days drug esposure. (TIF) [file pone.0046891.s003.tif]

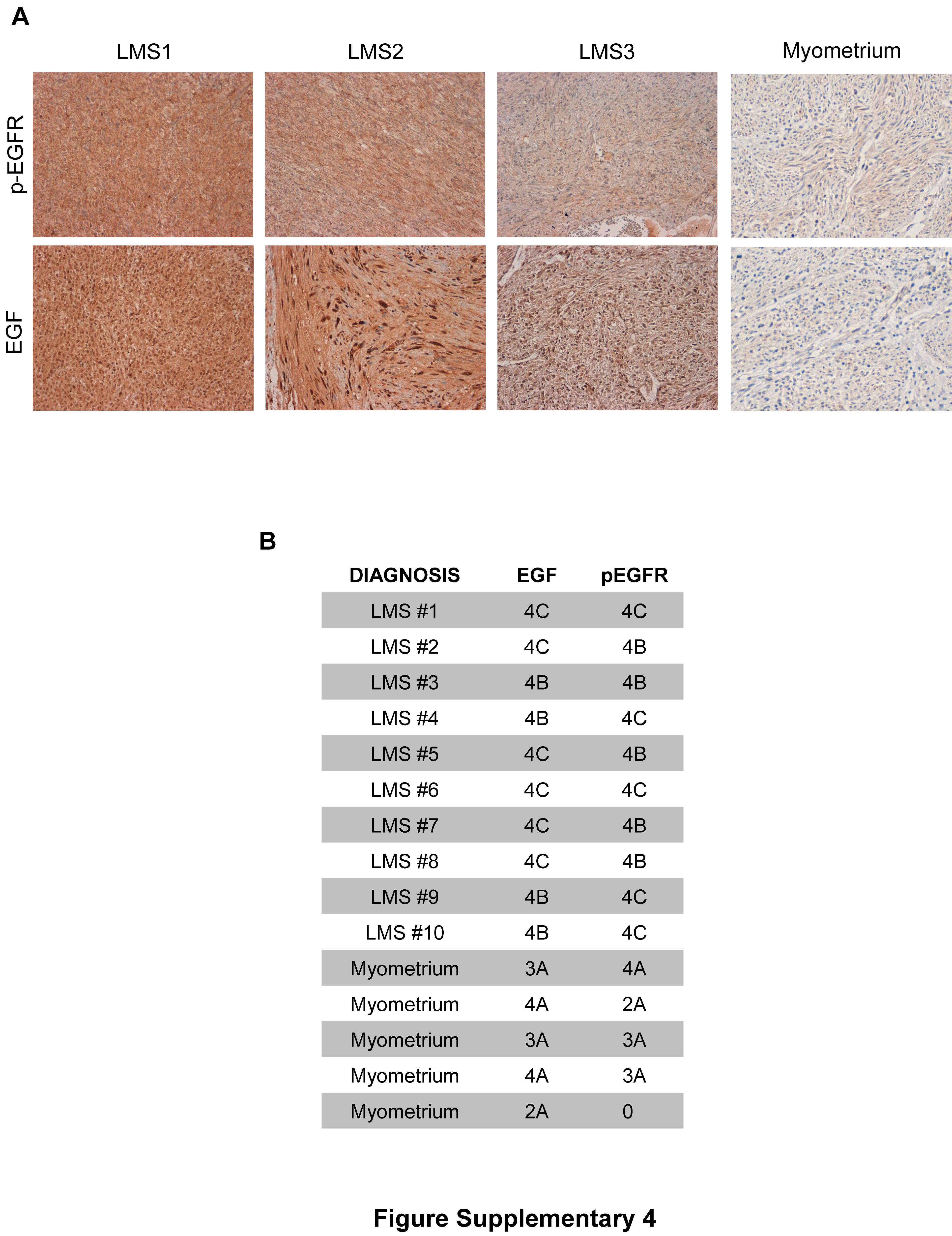

Supplement: Figure S4 — EGF/EGFR pathway is generally activated in leiomyosarcomas. A) p-EGFR and EGF immunohistochemistry in 3 out of 10 representative patient-derived LMS specimens. B) Table showing the EGF and pEGFR expression in 10 LMS patient-derived specimens and in 5 non tumoral tissue specimen (Myometrium). Values 1 to 4 indicating the percentage of positive cells with 0 = negative, 1<10%; 2 = 10–25%; 3 = 25–50%, 4<50% and letters A to C indicating the intensity of expression (A = weak,; B = moderate; C = high intensity). (TIF) [file pone.0046891.s004.tif]

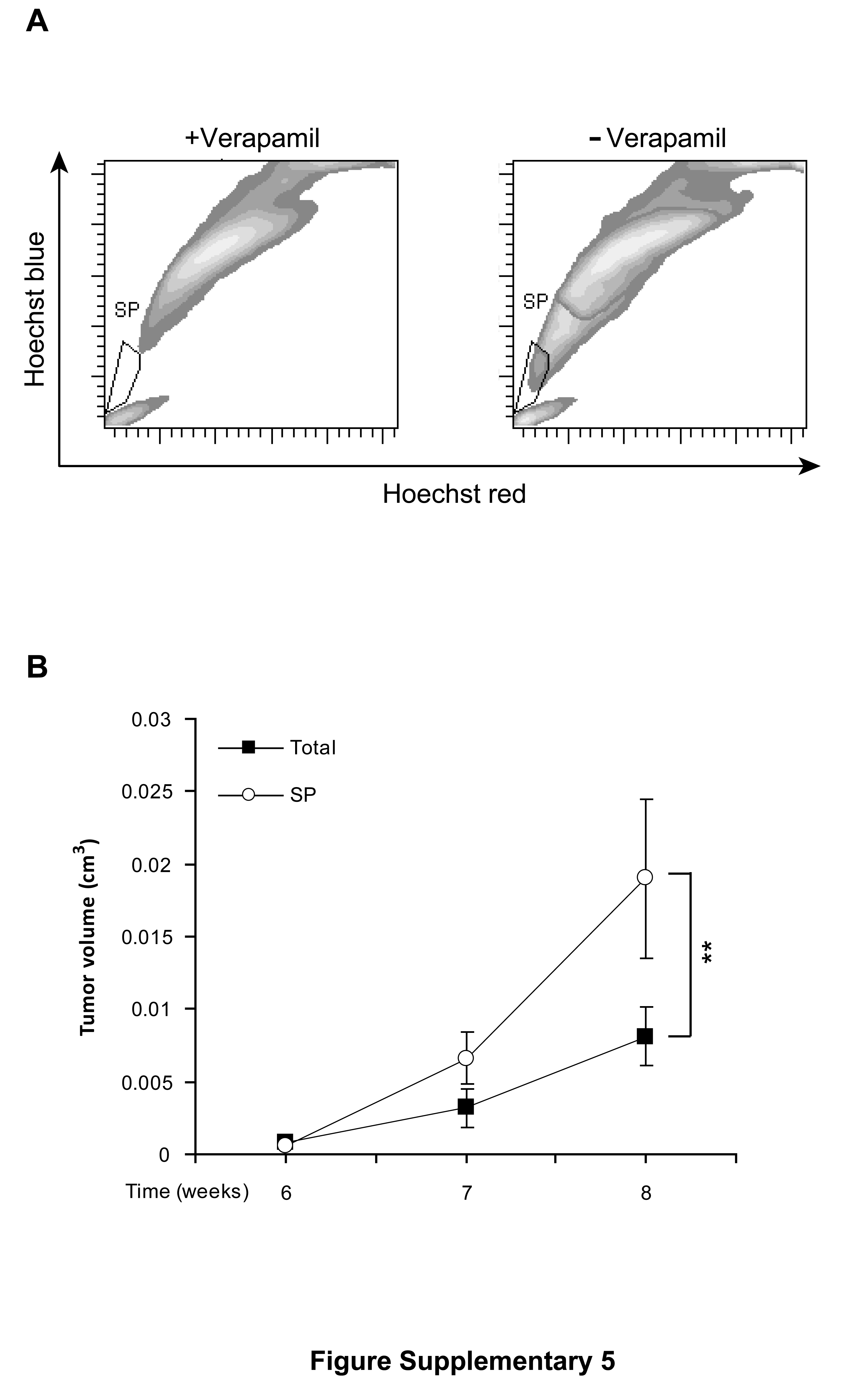

Supplement: Figure S5 — A) Cytofluorimetric cell sorting of side population (SP) cells (right panel). B) Tumor growth rate of xenografts generated by subcutaneous injection of sorted SP cells and unsorted undifferentiated LMS cells (adherent cultures). The values represent mean +/− SD of three independent experiments. Student’ s T test was used to determine p-value. **p<0,01. (TIF) [file pone.0046891.s005.tif]
